# Parent-Child Dyadic Coping and Quality of Life in Chronically Diseased Children

**DOI:** 10.3389/fpsyg.2021.701540

**Published:** 2021-07-28

**Authors:** Merel M. Nap-van der Vlist, Reine C. van der Wal, Eva Grosfeld, Elise M. van de Putte, Geertje W. Dalmeijer, Martha A. Grootenhuis, Cornelis K. van der Ent, Marry M. van den Heuvel-Eibrink, Joost F. Swart, Guy Bodenmann, Catrin Finkenauer, Sanne L. Nijhof

**Affiliations:** ^1^Department of Pediatrics, Wilhelmina Children’s Hospital, University Medical Center Utrecht, Utrecht University, Utrecht, Netherlands; ^2^Faculty of Social and Behavioural Sciences, Utrecht University, Utrecht, Netherlands; ^3^Julius Center for Health Sciences and Primary Care, University Medical Center Utrecht, Utrecht University, Utrecht, Netherlands; ^4^Princess Máxima Center for Pediatric Oncology, Utrecht, Netherlands; ^5^Department of Pediatric Pulmonology, Wilhelmina Children’s Hospital, University Medical Center Utrecht, Utrecht University, Utrecht, Netherlands; ^6^Department of Pediatric Rheumatology/Immunology, Wilhelmina Children’s Hospital, University Medical Center Utrecht, Utrecht University, Utrecht, Netherlands; ^7^Department of Psychology, University of Zurich, Zurich, Switzerland

**Keywords:** childhood disease, dyadic coping, parent-child relationship, quality of life, stress communication

## Abstract

Different forms of dyadic coping are associated with positive outcomes in partner relationships, yet little is known about dyadic coping in parent-child relationships. The current research explored the association between parent-child dyadic coping and children’s quality of life in 12–18-year old children with a chronic disease (i.e., cystic fibrosis, autoimmune diseases, and children post-cancer treatment). In a sample of 105 parent-child dyads, self-reported forms of dyadic coping (i.e., stress communication, problem-oriented, emotion-oriented, and negative dyadic coping) and children’s quality of life were assessed. Children reported more stress communication and negative dyadic coping than their parents, while parents reported more problem-oriented dyadic coping and emotion-oriented dyadic coping than their children. More stress communication of the child was associated with more emotion-oriented dyadic coping and less negative dyadic coping of the parent. More negative dyadic coping of the child was associated with less stress communication, problem-oriented dyadic coping and emotion-oriented dyadic coping of the parent. Additionally, both children’s and parents’ negative dyadic coping were associated with lower self-reported pediatric quality of life and parents’ emotion-oriented dyadic coping was associated with higher pediatric quality of life. These findings emphasize that children and their parents mutually influence each other and that dyadic coping is associated with children’s quality of life. Theoretical and practical implications are discussed.

## Introduction

Over the last decades, treatments for childhood diseases have advanced tremendously. Although the high survival rate for these children is indisputable a positive development, it also entails that more and more children grow up with a chronic disease (Verwey-Jonker Instituut, 2019). When growing up with a chronic disease, children encounter challenges not experienced by their healthy peers, including somatic aspects of the disease and psychosocial distress related to the disease (Perrin et al., 2007; Pinquart and Shen, 2011a,b; Maurice-Stam et al., 2019). The quality of the parent-child relationship is crucial in facing these challenges (Logan and Scharff, 2005; Cousino and Hazen, 2013; Pinquart, 2013; Palermo et al., 2014), as children often turn to their parents for increased support during stressful times. Yet, parents too are likely to experience extra stress, and this may impair their ability to provide adequate care for their child. In order to guide children and parents toward adaptive ways of coping with children’s chronic disease, it is important that parents and children jointly cope with stress, a process that is called dyadic coping (Bodenmann, 1997, 2005). The present study focusses on parent-child dyadic coping and quality of life in children with a chronic disease.

Dyadic coping involves the process in which two people in a relationship support each other and cope with a common stressor (Bodenmann, 1997, 2005). It represents a relational, hence dyadic, process because one partner’s coping behavior affects the other’s coping behavior and vice versa. Although several forms of dyadic coping may mitigate the negative effects of stress, such as stress communication, problem-oriented dyadic coping, and emotion-oriented dyadic coping, it may also actually increase stress when it is negative (Falconier and Kuhn, 2019). To illustrate, whereas offering emotional support in response to another person’s stress (i.e., emotion-oriented dyadic coping) may reduce subsequent stress, withdrawing from it (i.e., negative dyadic coping) may actually increase a person’s level of stress.

When families are confronted with stressful situations, such as a child’s chronic disease, it is crucial to consider children’s and parents’ mutual influence (Minuchin, 1985; Berg et al., 2009; Palermo et al., 2014). Indeed, when the child or one of the family members has a disease, it impacts the quality of life of all family members (Logan and Scharff, 2005; Cousino and Hazen, 2013; Pinquart, 2013; Palermo et al., 2014; Leeman et al., 2016). This suggests that the way in which children and parents cope with a child’s chronic disease is not only affected by the disease-related stress they experience themselves, but also by the stress they perceive in and from each other.

Various studies among romantic couples have demonstrated that the way and quality of couples’ dyadic coping correlate with levels of psychological well-being and relationship quality, also for couples in which one of the partners had a disease, such as cystic fibrosis or breast cancer (Rottmann et al., 2015; Falconier and Kuhn, 2019; Werner et al., 2020). Studies in patients with chronic obstructive pulmonary disease or myocardial infarction and their partners have shown that some forms of dyadic coping, such as stress communication or active engagement lead to higher quality of life of the person coping with a medical condition (Joekes et al., 2007; Vaske et al., 2015). In addition, more negative dyadic coping, as well as lower levels of supportive dyadic coping, have shown to be associated with more distress of cardiac patients (Rapelli et al., 2021). Also, dyadic coping plays a role in parents’ individual and relational outcomes and family adjustment, for example when they face a cancer diagnosis of their child (Van Schoors et al., 2019b,c).

Yet, it is important to note that research on dyadic coping almost exclusively focused on romantic relationships (Falconier et al., 2015), in which members have a stable equal standing. Dyadic coping may work differently in parent-child relationships, which are less reciprocal and more unequal. Specifically, parents have the responsibility to stimulate their child’s autonomy and competence, while providing safety and attachment. Children are not expected to reciprocate the provision of safety and attachment for their parents (e.g., Laursen and Collins, 2009). Moreover, parents have higher standing, in terms of authority, responsibility, life-experience, and knowledge than their children. As children grow older, the form and extent to which the parent-child relationship is unequal changes. For example, although the parent-child relationship remains unequal, when children enter adolescence they gradually depend less on their parents and their autonomy grows (e.g., Branje et al., 2013; Smetana and Rote, 2019). This increased (search for) autonomy may go hand in hand with more negative forms of dyadic coping. Older versus younger adolescents may communicate less about their negative feelings regarding the disease to their parents, which may affect parents’ coping responses accordingly. Hence, in order to explore dyadic coping in parent-child relationships, it is important to take the child’s age into account.

Chronic pediatric disease offers a unique context to study the association between different forms of parent-child dyadic coping and children’s quality of life. Although there is a broad range of childhood chronic diseases, the psychosocial impact of growing up with an ever-present disease shows considerable similarities across various diseases (Stein and Jessop, 1989). For example, almost all children experience the burden of a treatment regime, restrictions in daily life activities, and stress surrounding doctor’s visits. Parents may experience more parenting stress, more psychological distress, and perhaps more problems with family functioning (Logan and Scharff, 2005; Cousino and Hazen, 2013; Pinquart, 2013; Palermo et al., 2014; Leeman et al., 2016). In the current research, we therefore investigated a broad range of childhood chronic diseases; particularly cystic fibrosis, autoimmune diseases and children who face long-term consequences after the treatment of childhood cancer.

The present study had two primary aims and a few secondary aims. Our first primary aim was to explore whether and how children with a chronic disease and their parents engage in dyadic coping. Our second primary aim was to explore how different forms of dyadic coping are associated with children’s quality of life. We did not have *a priori* expectations whether parents and children would use different forms of dyadic coping. We did expect, however, (1) that less negative dyadic coping of the child would be associated with higher self-reported quality of life, and (2) that more stress-communication, problem-oriented dyadic coping and emotion-oriented dyadic coping of the parent would be associated with higher quality of life of the child.

Our secondary aims were to explore whether the age of the child affected parent-child dyadic coping, and whether parent-child dyadic coping differs depending on the family role (father versus mother), family status (non-intact versus intact), and disease group. Due to the increase in (need for) autonomy during adolescence, we expected that older children would demonstrate less stress communication, problem-oriented, and emotion-oriented dyadic coping. We also expected that parents and children in a non-intact family may experience more stress, which may amplify the dyadic coping and quality of life associations. Finally, given the diverse sample and explorative character of the study, we explored differences in parent-child dyadic coping regarding the family role and disease group.

## Materials and Methods

### Participants

This study used a sample of patients of the Wilhelmina Children’s Hospital and the Princess Máxima Center for Pediatric Oncology in the Netherlands and one of their parents, recruited from January 2018 through November 2020. This cross-sectional data was part of the larger PROactive cohort study with annual measurements with chronically ill patients and their families (Nap-van der Vlist et al., 2019). The goal of the larger study was explore fatigue, well-being and associated factors in children with a range of pediatric chronic diseases (Nap-van der Vlist et al., 2019). Children are included at least 1 year after diagnosis (CF/autoimmune disease) or after the completion of treatment for childhood cancer. This study was based on an extended follow-up assessment including children around 12 (*N* = 32), fifteen (*N* = 42), or eighteen (*N* = 31) years of age and one of their parents. In order to minimize the burden for the family, only one parent was asked to participate, to the preference of the family. The study was classified by the institutional review board as exempt of the Medical Research Involving Human Subjects Act (16-707/C) and adhered to all local laws and the declaration of Helsinki.

For this study, 255 families were approached, of which 110 parent-child dyads participated (43.1% participation rate). We removed five dyads in which parents did not complete the questionnaire within the same month as their child, reducing our total sample to 105 dyads. Children (60 girls; 57%) had a mean age of 15.43 years (*SD* = 2.27; range 12–19 years). Parents (84 mothers; 80%) had a mean age of 47.49 years (*SD* = 4.92). In total, 19 children were from non-intact families (18%) (i.e., long or permanent separation of the parents, divorce, or death of a parent). Twenty-seven children suffered from cystic fibrosis (CF), 58 from an autoimmune disease (e.g., Juvenile Idiopathic Arthritis (JIA), and 20 were involved in early post-cancer treatment (median follow-up time 1.9 years since diagnosis). Notably, we did not find any significant differences in participating and non-participating children’s sex or disease group, except for children’s age. Non-participants were slightly older than participants (*Mean* = 15.98 versus *Mean* = 15.35 years old, *p* = 0.03).

### Procedure

Before an outpatient visit, the researchers approached families via email to take part in the study. If they agreed to participate, formal informed consent was obtained from children and one parent. They completed the questionnaires via a web-based tool^1^ separately from each other. All participants received—if necessary—one reminder to participate in the study via email and one via telephone. A research team was available to answer questions via email and telephone.

### Measures

#### Dyadic Coping

To explore the concept of parent-child dyadic coping, we measured different forms of dyadic coping with five items from the Dyadic Coping Inventory, which originally contains 37 items (Bodenmann, 2008). Children and parents were asked how they generally respond to stressful situations. Children were asked which parent they spent most time with and were asked to fill out the dyadic coping question with this parent in mind. We only included dyads in which the parent who filled out the questionnaire was the same parent as the child had taken in mind. We adapted the items for the parent-child relationship, which covered four sub dimensions of dyadic coping (Supplementary Table 1): (1) stress communication; “I show my father/mother/child when I am not doing well or when I have problems,” and “I tell my father/mother/child openly how I feel and that I need his/her support,” (2) problem-oriented dyadic coping; “When my father/mother/child is stressed out, I give him/her good advice or practical help,” (3) emotion-oriented dyadic coping; “I listen to my father/mother/child so that he/she can tell what really bothers him/her,” and (4) negative dyadic coping; “When my father/mother/child is stressed, I tend to withdraw.” All items were measured on a scale from 1 (*very rarely*) to 5 (*very often*). The two stress communication items correlated strongly with each other (*r* = 0.70 for children, and *r* = 0.65 for parents), we therefore took the mean of these two items as indicator of stress communication.

#### Quality of Life

Children reported on their own quality of life with the validated Pediatric Quality of Life Generic Core Scale 4.0 (PedsQL GCS; Engelen et al., 2009) consisting of 23 items measured on a scale from 0 (*never a problem*) to 4 (*almost always a problem*). The PedsQL GCS covers the domains physical functioning, emotional functioning, social functioning, and school functioning. Only the total score was used. Example items were “It is hard for me to do sports activity or exercise” and “It is hard to keep up with my peers.” Answers were reverse-coded to a scale from 0 to 100, such that higher scores indicated a higher quality of life. Cronbach’s α of all items for the total-score was .83.

#### Clinical Characteristics

In order to characterize our sample, we gathered information regarding the time elapsed between diagnosis and assessment, and disease activity at assessment from the child’s medical record. Disease-specific characteristics were not included in the analyses but used to describe that this was a sample in which the disease activity was generally low or absent (Supplementary Table 2). In CF, disease activity was measured using forced expiratory volume in one second (FEV_1_), expressed as percentage of predicted FEV_1_ (Quanjer et al., 2012). For JIA, the validated clinical Juvenile Arthritis Disease Activity Score (cJADAS) and erythrocyte sedimentation rate (ESR) were used as a proxy for disease status; for participants with other autoimmune diseases, ESR was used (McErlane et al., 2016). All children post-cancer treatment finished their treatment protocols and were in complete remission.

### Statistical Analyses

We conducted our analyses in RStudio (RStudio Team, 2019). We first explored mean differences between parents and children on their dyadic coping responses by conducting paired *t*-tests. We additionally explored the effects of the age of the child on parent-child dyadic coping, by conducting a one-way MANOVA analysis with age group as fixed factor and the dyadic coping responses as dependent variables. To address the question whether and how different forms of dyadic coping were associated with children’s quality of life we conducted Spearman correlations, since some of the study variables (i.e., children’s problem-oriented dyadic coping and quality of life, and parents’ stress communication, problem-oriented dyadic coping, and negative dyadic coping) were non-normally distributed. In Supplementary Table 3, we reported the correlation analyses controlling for children’s age. Although the small sample sizes of the subgroups did not allow us to draw firm conclusions, we also explored whether family status (non-intact versus intact), family role (father or mother), and disease group revealed different dyadic coping responses. We therefore conducted a one-way full factorial MANOVA analysis with the sub-groups as fixed factors and the dyadic coping responses as dependent variables (Supplementary Table 4).

## Results

### Differences in Parent-Child Dyadic Coping Between Parents and Children

Children reported significantly more stress communication and negative dyadic coping than their parents, while parents reported significantly more problem-oriented dyadic coping and emotion-oriented dyadic coping than their children (Table 1). Moreover, there was a statistically significant difference in dyadic coping responses by children depending on the age group of the child, *F*(16, 190) = 1.70, *p* = 0.050; Wilk’s Λ = 0.766, partial η^2^ = 0.13. Specifically, 12-year old children reported significantly higher levels of stress communication than 15-year and 18-year old children (Table 2).

**TABLE 1:** Means and standard deviations of main study variables in the overall sample.

	Child		Parent		*t*	*df*	*p*
	*M*	*SD*	*M*	*SD*			
1. Stress communication	3.94	0.81	2.98	0.71	–9.279	104	<0.001
2.Problem-oriented dyadic coping	3.39	0.84	3.99	0.69	5.743	104	<0.001
3.Emotion-oriented dyadic coping	4.03	0.77	4.24	0.56	2.427	104	0.017
4. Negative dyadic coping	2.56	1.10	1.90	0.87	–4.934	104	<0.001
5.Quality of life	77.87	16.14					

*n = 105 parent-child dyads. The dyadic coping items are scored on a scale from 1 (very rarely) to 5 (very often). Quality of life was scored with the total score of the PedsQL GCS on a scale from 0 to 100, with a higher score indicating higher quality of life.*

*M, Mean; SD, standard deviation.*

**TABLE 2:** Means and standard deviations of different forms of dyadic coping per age group.

	12 years (*n* = 32)		15 years (*n* = 42)		18 years (*n* = 31)		*df*	*F*	*p*	η^2^
	*M*	*SD*	*M*	*SD*	*M*	*SD*				
**Child**										
1. Stress communication	4.33^a^	0.78	3.77^b^	0.83	3.77^b^	0.71	2, 102	5.78	0.004	0.10
2. Problem-oriented dyadic coping	3.47	0.98	3.21	0.87	3.55	0.57	2, 102	1.64	0.199	0.03
3. Emotion-oriented dyadic coping	4.03	0.90	3.93	0.75	4.16	0.64	2, 102	0.82	0.442	0.02
4. Negative dyadic coping	2.34	1.23	2.45	1.06	2.94	0.93	2, 102	2.71	0.071	0.05
**Parent**										
6. Stress communication	2.95	0.90	3.05	0.56	2.90	0.68	2, 102	0.39	0.678	0.01
7. Problem-oriented dyadic coping	3.97	0.74	4.07	0.56	3.90	0.79	2, 102	0.55	0.576	0.01
8. Emotion-oriented dyadic coping	4.28	0.58	4.24	0.53	4.19	0.60	2, 102	0.19	0.829	0.004
9. Negative dyadic coping	1.75	0.92	1.86	0.75	2.10	0.94	2, 102	1.34	0.266	0.03

*The dyadic coping items are scored on a scale from 1 (very rarely) to 5 (very often).*

*M, Mean; SD, standard deviation.*

*^*a,b*^Means that do not share superscripts differ within the subcategories of the rows.*

### Correlations Parent-Child Dyadic Coping and Children’s Quality of Life

Within the child sample, stress communication, problem-oriented dyadic coping and emotion-oriented dyadic coping were positively associated with each other, and negatively with negative dyadic coping. This suggests that different forms of dyadic coping may reinforce each other and mitigate negative dyadic coping. The same was true for the parent sample, although the effects were less strong (Table 3).

**TABLE 3:** Correlation matrix of main study variables.

	1	2	3	4	5	6	7	8
**Child**								
1. Stress communication								
2. Problem-oriented dyadic coping	0.39***							
3. Emotion-oriented dyadic coping	0.49***	0.41***						
4. Negative dyadic coping	−0.30**	−0.23*	–0.15					
5. Quality of life	0.10	0.13	0.08	−0.41***				
**Parent**								
6. Stress communication	0.02	0.10	0.13	−0.21*	0.13			
7. Problem-oriented dyadic coping	0.07	0.08	0.08	−0.22*	0.12	0.27**		
8. Emotion-oriented dyadic coping	0.21*	0.22*	0.18	−0.26**	0.26**	0.26**	0.51***	
9. Negative dyadic coping	−0.21*	–0.18	0.01	0.04	−0.19*	0.08	–0.08	−0.21*

n = 105 parent-child dyads. *p < 0.05. **p < 0.01. ***p < 0.001.

Moreover, children’s dyadic coping was associated with their parents’ dyadic coping in several ways. More stress communication of the child was associated with more emotion-oriented dyadic coping and less negative dyadic coping of the parent. More negative dyadic coping of the child was associated with less stress communication, less problem-oriented dyadic coping, and less emotion-oriented dyadic coping of the parent. More problem-oriented dyadic coping of the child was associated with more emotion-oriented dyadic coping of the parent.

The form in which parents and children with a chronic disease dyadically coped was associated with children’s quality of life. In particular, both children’s and parents’ negative dyadic coping were associated with a lower quality of life of the children. Parents’ emotion-oriented dyadic coping was associated with a higher quality of life of children. Importantly, controlling for children’s age did not change the pattern of results. The significant associations only became slightly weaker (see Supplementary Table 3).

### Differences in Parent-Child Dyadic Coping Between Subgroups

The one-way MANOVA did not reveal significant main effects of family situation, family role, or disease group, nor did it reveal any interaction effects, all *F*’s < 1.55, and *p*’s > 0.15 (Supplementary Table 4).

## Discussion

The current research is the first to our knowledge to explore dyadic coping in parent-child relationships and its association with children’s quality of life in a population of children with a chronic disease. Regarding our primary aims, children reported more stress communication and negative dyadic coping (i.e., withdrawing and avoiding the parent’s stress) than their parents, while parents reported more problem-oriented dyadic coping (i.e., offering practical support) and emotion-oriented dyadic coping (i.e., showing emotional interest and empathy, caring) than their children. More stress communication of the child was associated with more emotion-oriented dyadic coping and less negative dyadic coping of the parent (i.e., withdrawing and avoiding from the child’s stress). More negative dyadic coping of the child was also associated with less stress communication, problem-oriented dyadic coping and emotion-oriented dyadic coping of the parent. As expected, parents’ emotion-oriented dyadic coping was associated with higher pediatric quality of life and parents’ negative dyadic coping was associated with lower pediatric quality of life. The child’s own problem-oriented (i.e., offering his/her parent practical support) and emotion-oriented (i.e., showing emotional interest in his/her parent) dyadic coping was not related to their quality of life, except for their own negative dyadic coping that yielded a relatively strong negative association. The more they engage in negative dyadic coping the lower their quality of life, reflecting that either this negativity toward their parents diminishes their own well-being or their negative dyadic coping mirrors a healthy adolescent behavior to withdraw when parents overburden them with their stress. Latter explanation seems more convincing as the means of stress communication, problem-oriented dyadic coping, and emotion-oriented dyadic coping show a relatively normal level of engagement in dyadic stress management (Ledermann et al., 2010). An alternative explanation is that children might reciprocate the negative dyadic coping received from parents, in turn negatively impacting children’s quality of life (Donato and Parise, 2012). It is also noteworthy that children with a chronic illness reported high scores in own stress communication toward their parents (while those on the other hand scored noticeably lower, probably in order not to burden their child or by respecting generational borders) that speaks for a certain openness toward their parents. This yields an interesting picture. On the one hand they talk about their stress to their parents, feel better (higher quality of life) when they receive emotional supportive dyadic coping and at the same time engage in more negative dyadic coping. Perhaps, this pattern is linked to age, as was explored as one of the secondary aims. The older the children (in the phase of adolescence) the less they express their stress to their parents and the more they engage in negative dyadic coping, possibly standing for a search of autonomy. Perhaps, parents may react on this delimitation of the child by suppressing their own stress communication, problem- and emotion-oriented dyadic coping. Overall, these findings may reflect how children and their parents try to find a balance between stress communication and dyadic coping on the one hand and autonomy on the other hand.

Important contributions of the present study are its focus on dyadic coping processes specifically (a) in parent-child relationships, (b) in the context of a chronic illness and (c) that fact that these processes are embedded in different phases of adolescence, from pre-adolescence to late adolescence. In previous studies, dyadic coping was generally investigated in romantic adolescent or adult relationships (Falconier et al., 2015; Breitenstein et al., 2018; Bodenmann et al., 2019; Van Schoors et al., 2019a) and has not considered parent-child relationships. Albeit, general dyadic processes, but not *dyadic coping*, have been studied in parent-child relationships. For example, the effect of parental and pediatric behavior, such as how parents and children talk to one another, on pediatric outcomes has been studied (Birnie et al., 2016; Neville et al., 2020). Extending these findings, our results suggest that especially children’s and parents’ negative dyadic and parents’ emotion-oriented dyadic coping are important indicators for a child’s quality of life. These findings correspond with other studies showing the negative outcomes and ineffectiveness of avoidant coping strategies in response to stress (Compas et al., 2012), and the positive effects of an open climate in the family (Niemi, 1988; Lutz et al., 2007; Meriggi et al., 2017). Communicating stress might be a first step in providing good dyadic coping (more emotion-oriented coping and less negative coping). At the same time, and most importantly, these findings highlight that the way in which children with a chronic disease and their parents cope with stress is not only affected by the stress they experience themselves, but also by the stress of the other, the context (adolescence) and the generational affiliation. Despite this complex framework (chronic illness, parent-child, adolescence), dyadic coping proved to be an important variable for child’s quality of life. This is an important, novel and promising finding, which opens up a new field of research for psychologists, medical scientists and nursing staff or general caregivers. It implies that in order to understand factors and processes associated with children’s quality of life, we need to consider the social context in which children grow up.

### Limitations and Future Research Directions

The objective of this study was to highlight that parent-child dyadic coping can have an effect on the child’s well-being in the context of chronic pediatric disease. A first limitation of this study is that we used a limited number of selected items of a preexisting questionnaire, validated in the context of close relationships. An important starting point for future research following this limitation is to develop and validate a new dyadic coping questionnaire or to adapt the Dyadic Coping Inventory (DCI) assessing coping processes among parents and children. This questionnaire should be tested in healthy populations as well as in children with a chronic disease and their parents, in order to explore the differences in dyadic coping between these groups. Such a validated questionnaire is required to examine dyadic associations in which children’s and parents’ dyadic coping may influence both children’s and parents’ quality of life or other outcomes relevant to families coping with and adjusting to disease (i.e., dyadic data analysis). The use of dyadic analytical technics in the future could provide a more comprehensive understanding of parent-child dyads as an interactional unit. To further improve the generalization of the findings, future work should take into account parent-child dyads varying in demographic information (i.e., education, ethnicity). Another limitation is that the DCI asks how children and parents deal with common stressors, of which the disease is one, but of course the DCI measures stressors broader than the disease alone. Nevertheless, as parents and children with a chronic disease encounters extra stressors compared to healthy parent-child dyads, supporting them in dyadic coping is still of interest. A final limitation relates to the statistical analyses that were merely of exploratory nature. Longitudinal data is necessary to illuminate directionality of the associations between parent-child dyadic coping and children’s quality of life.

Future research might also tap into underlying mechanisms explaining the association between parent-child dyadic coping and children’s quality of life. Children and parents face the challenge of managing their own stress as well as communicating about this stress and reacting to the other person’s stress. The dynamics of communicating stress and managing one’s own stress are worth investigating. Also, as it was shown that protective buffering in parent-child dyads was associated with reduced authenticity, one possibility is that parents’ and children’s negative dyadic coping is associated with reduced feelings of authenticity, which in turn may hamper children’s quality of life (van der Wal et al., 2021). Last, perceptions of the other person’s dyadic coping may affect children’s and parents’ outcomes to a greater extent than one’s own dyadic coping. To illustrate, the way in which children perceive their parent is responding to their stress (for example, by withdrawing) may not correspond to the way in which parents themselves think they are coping with the stress of their child. Indeed, research shows that the support given by one dyad member is not always seen as positive support by the other dyad member (Fales et al., 2014) and that equity in the contributions matter particularly (Meier et al., 2020). Therefore, it is important in a next study to use the complete DCI to compare the different appraisals of dyadic coping by the various protagonists.

## Conclusion

In conclusion, the findings of our explorative, dyadic study add to the small body of work on dyadic processes in parent-child relationships, and in particular in the context of a child’s chronic disease. The results highlight that children and their parents mutually influence each other and that both parents’ and children’s negative dyadic coping is associated with lower self-reported pediatric quality of life and parents’ emotion-oriented dyadic coping is associated with higher pediatric quality of life, although the correlational nature of the data prohibit causal inferences. It provides further impetus for studies aimed at understanding and promoting adaptive dyadic coping of parents and children facing stress. Helping children and their parents to jointly deal with stressful situations, such as children’s chronic disease, may not only empower children and benefitting the child’s quality of life but it may also improve the parent’s quality of life. The extent to which parent-child dyadic coping affect children’s functioning is an intriguing and (yet) unanswered question. We hope that the current findings offer a springboard to further explore the role of parent-child dyadic coping in children with a chronic disease.

## Data Availability Statement

The datasets presented in this article are not readily available because the informed consent stated that the data of participants would not be shared with third parties. Therefore, our data cannot be made publicly available. Requests to access the datasets should be directed to SN, s.l.nijhof@umcutrecht.nl.

## Ethics Statement

The studies involving human participants were reviewed and approved by the institutional review board as exempt of the Medical Research Involving Human Subjects Act (16-707/C) and adhered to al local laws and the declaration of Helsinki. Written informed consent to participate in this study was provided by the participants’ legal guardian/next of kin.

## Author Contributions

MN-V, RW, and EG conducted the analyses and wrote the drafts of the manuscript. CF, SN, EP, GD, MG, CE, MH-E, JS, and GB critically reviewed the data analyses and reviewed the manuscript for important intellectual content. MN-V, RW, CF, SN, and EP developed the study. MN-V and SN obtained approval from the ethical committee and collected the data. All authors contributed to the article and approved the submitted version.

## Conflict of Interest

The authors declare that the research was conducted in the absence of any commercial or financial relationships that could be construed as a potential conflict of interest.

## Publisher’s Note

All claims expressed in this article are solely those of the authors and do not necessarily represent those of their affiliated organizations, or those of the publisher, the editors and the reviewers. Any product that may be evaluated in this article, or claim that may be made by its manufacturer, is not guaranteed or endorsed by the publisher.

## Footnotes

1
www.hetklikt.nu
