# Supplementary material for: Parent-Child Dyadic Coping and Quality of Life in Chronically Diseased Children
Source: Front Psychol. 2021 Jul 28;12:701540. doi: 10.3389/fpsyg.2021.701540 (PMC8355494; doi:10.3389/fpsyg.2021.701540)
Supplement: Supplementary file 1 [file Data_Sheet_1.docx]

Supplementary Material

Supplementary Table S1. Overview of the included dyadic coping items for children and their parents

| **Item** | **Parent** | **Child** |
| --- | --- | --- |
| Stress communication | “I show my child when I am not doing well or when I have problems”, and “I tell my child openly how I feel and that I need his/her support” | “I show my father/mother when I am not doing well or when I have problems”, and “I tell my father/mother openly how I feel and that I need his/her support” |
| Problem-oriented dyadic coping | “When my child is stressed out, I give him/her good advice or practical help” | “When my father/mother is stressed out, I give him/her good advice or practical help” |
| Emotion-oriented dyadic coping | “I listen to my child so that he/she can tell what really bothers him/her” | “I listen to my father/mother so that he/she can tell what really bothers him/her” |
| Negative dyadic coping | “When my child is stressed, I tend to withdraw” | “When my father/mother is stressed, I tend to withdraw” |

*Note. Items adapted from the Dyadic Coping Inventory* (Bodenmann, 2008)*.*

Supplementary Table S2. Children’s disease-specific characteristics

|  | **Cystic fibrosis (*n* = 27)** | **Autoimmune disease (*n* = 58)** | **Post-cancer treatment (*n* = 20)** |
| --- | --- | --- | --- |
| Diagnosis | 16 (59%) homozygote dF508  10 (37%) heterozygote dF508  1 (4%) other | 11 (19%) poly-articular JIA  24 (43%) oligo-articular JIA  3 (7%) systemic JIA  6 (10%) other form of JIA  8 (10%) immunodeficiency  3 (2%) auto-inflammatory condition  3 (10%) systemic auto-immune disease | 10 (43%) solid tumors  9 (43%) leukemia/lymphoma  1 (14%) brain tumor |
| Disease duration* | 16.0 ± 2.3 | 8.5 ± 3.9 | 1.9 ± 0.8 |
| Disease activity | FEV_1_ %:  80.9 ± 18.1 | cJADAS: 0 (0-4) (*n* = 43)  ESR: 2 mm/1st hr (2-5) (*n* = 50) | All post-treatment |
| *Notes. JIA = juvenile idiopathic arthritis; cJADAS = clinical Juvenile Arthritis Disease Activity Score; ESR = erythrocyte sedimentation rate; FEV_1_ % = predicted percentage of forced expiratory volume in one second.* Disease duration: years since diagnosis until inclusion for children with JIA; years from end of treatment until inclusion for children post-cancer treatment. If the data were normally distributed, the mean ± SD are given; if not, the median and interquartile range (IQR) are given.* | | | |

Supplementary Table S3. Correlation matrix of the variables controlling for children’s age

|  | 1 | 2 | 3 | 4 | 5 | 6 | 7 | 8 |
| --- | --- | --- | --- | --- | --- | --- | --- | --- |
|  |  |  |  |  |  |  |  |  |
| **Child** |  |  |  |  |  |  |  |  |
| 1. Stress communication |  |  |  |  |  |  |  |  |
| 2. Problem-oriented dyadic coping | .47^***^ |  |  |  |  |  |  |  |
| 3. Emotion-oriented dyadic coping | .53^***^ | .45^***^ |  |  |  |  |  |  |
| 4. Negative dyadic coping | -.24^*^ | -.18 | -.12 |  |  |  |  |  |
| 5. Quality of life | .04 | .07 | .05 | -.37^***^ |  |  |  |  |
| **Parent** |  |  |  |  |  |  |  |  |
| 6. Stress communication | .02 | .07 | .05 | -.18 | .14 |  |  |  |
| 7. Problem-oriented dyadic coping | .01 | .02 | .06 | -.20^*^ | .05 | .27^**^ |  |  |
| 8. Emotion-oriented dyadic coping | .20^*^ | .19 | .14 | -.24^*^ | .22^*^ | .25^**^ | .50^***^ |  |
| 9. Negative dyadic coping | -.19 | -.19 | -.06 | -.02 | -.21^*^ | .06 | -.04 | -.18 |

*Notes. n = 105 parent-child dyads. ^*^p < .05. ^**^p < .01. ^***^p < .001.*

Supplementary Table S4. Means and standard deviations of main study variables per family situation, family role, and disease group

|  | **Family situation** | | | | **Family role** | | | | **Disease group** | | | | | |
| --- | --- | --- | --- | --- | --- | --- | --- | --- | --- | --- | --- | --- | --- | --- |
|  | Intact  (*n* = 86) | | Non-intact  (*n* = 19) | | Mother  (*n* = 84) | | Father  (*n* = 21) | | CF  (*n* = 27) | | AI  (*n* = 58) | | PC  (*n* = 20) | |
|  | *M* | *SD* | *M* | *SD* | *M* | *SD* | *M* | *SD* | *M* | *SD* | *M* | *SD* | *M* | *SD* |
| **Child** |  |  |  |  |  |  |  |  |  |  |  |  |  |  |
| 1. Stress communication | 3.92 | 0.82 | 4.03 | 0.81 | 3.94 | 0.83 | 3.93 | 0.76 | 3.94 | 0.71 | 3.90 | 0.88 | 4.05 | 0.78 |
| 2. Problem-oriented dyadic coping | 3.41 | 0.89 | 3.32 | 0.58 | 3.39 | 0.85 | 3.38 | 0.81 | 3.41 | 0.69 | 3.36 | 0.85 | 3.45 | 0.99 |
| 3. Emotion-oriented dyadic coping | 4.01 | 0.78 | 4.11 | 0.74 | 4.00 | 0.78 | 4.14 | 0.73 | 4.07 | 0.73 | 4.00 | 0.77 | 4.05 | 0.83 |
| 4. Negative dyadic coping | 2.48 | 1.14 | 2.95 | .85 | 2.57 | 1.09 | 2.52 | 1.17 | 2.70 | 1.03 | 2.47 | 1.20 | 2.65 | .88 |
| **Parent** |  |  |  |  |  |  |  |  |  |  |  |  |  |  |
| 6. Stress communication | 2.94 | 0.70 | 3.13 | 0.76 | 2.92 | 0.67 | 3.21 | 0.82 | 3.06 | 0.74 | 2.93 | 0.65 | 3.00 | 0.86 |
| 7. Problem-oriented dyadic coping | 3.99 | 0.71 | 4.00 | 0.58 | 3.99 | 0.69 | 4.00 | 0.78 | 3.93 | 0.55 | 3.98 | 0.78 | 4.10 | 0.55 |
| 8. Emotion-oriented dyadic coping | 4.24 | 0.59 | 4.21 | 0.42 | 4.20 | 0.58 | 4.38 | 0.50 | 4.30 | 0.47 | 4.19 | 0.61 | 4.30 | 0.57 |
| 9. Negative dyadic coping | 1.85 | 0.85 | 2.11 | 0.94 | 1.88 | 0.90 | 1.95 | 0.74 | 2.26 | 1.06 | 1.81 | 0.81 | 1.65 | 0.59 |

*Notes. M = Mean; SD = standard deviation; CF = cystic fibrosis; AI = auto-immune disease; PC = post-cancer treatment.*
